# Psychiatric Symptoms in Wilson’s Disease—Consequence of *ATP7B* Gene Mutations or Just Coincidence?—Possible Causal Cascades and Molecular Pathways

**DOI:** 10.3390/ijms252212354

**Published:** 2024-11-18

**Authors:** Grażyna Gromadzka, Agnieszka Antos, Zofia Sorysz, Tomasz Litwin

**Affiliations:** 1 Department of Biomedical Sciences, Faculty of Medicine, Collegium Medicum, Cardinal Stefan Wyszynski University, Wóycickiego Street 1/3, 01-938 Warsaw, Poland; 2 Second Department of Neurology, Institute of Psychiatry and Neurology, Sobieskiego Street 9, 02-957 Warsaw, Poland; agantos@ipin.edu.pl; 3 Students Scientific Association “Immunis”, Cardinal Stefan Wyszynski University, Dewajtis Street 5, 01-815 Warsaw, Poland

**Keywords:** Wilson’s disease, psychiatric symptoms, molecular pathogenesis, psychosis, schizophrenia, bipolar disorder, cognitive disorders, oxidative stress, mitophagy, cuproptosis, ferroptosis, inflammation, immunological/autoimmunological processes, copper toxicity, neurotrophic factors, ncRNA, microRNA, circRNA, lncRNA

## Abstract

Wilson’s disease (WD) is an autosomal recessive disorder of copper metabolism. The genetic defect in WD affects the *ATP7B* gene, which encodes the ATP7B transmembrane protein, which is essential for maintaining normal copper homeostasis in the body. It is primarily expressed in the liver and acts by incorporating copper into ceruloplasmin (Cp), the major copper transport protein in the blood. In conditions of excess copper, ATP7B transports it to bile for excretion. Mutations in *ATP7B* lead to impaired ATP7B function, resulting in copper accumulation in hepatocytes leading to their damage. The toxic “free”—unbound to Cp—copper released from hepatocytes then accumulates in various organs, contributing to their damage and clinical manifestations of WD, including hepatic, neurological, hematological, renal, musculoskeletal, ophthalmological, psychiatric, and other effects. While most clinical manifestations of WD correspond to identifiable organic or cellular damage, the pathophysiology underlying its psychiatric manifestations remains less clearly understood. A search for relevant articles was conducted in PubMed/Medline, Science Direct, Scopus, Willy Online Library, and Google Scholar, combining free text and MeSH terms using a wide range of synonyms and related terms, including “Wilson’s disease”, “hepatolenticular degeneration”, “psychiatric manifestations”, “molecular mechanisms”, “pathomechanism”, and others, as well as their combinations. Psychiatric symptoms of WD include cognitive disorders, personality and behavioral disorders, mood disorders, psychosis, and other mental disorders. They are not strictly related to the location of brain damage, therefore, the question arises whether these symptoms are caused by WD or are simply a coincidence or a reaction to the diagnosis of a genetic disease. Hypotheses regarding the etiology of psychiatric symptoms of WD suggest a variety of molecular mechanisms, including copper-induced CNS toxicity, oxidative stress, mitochondrial dysfunction, mitophagy, cuproptosis, ferroptosis, dysregulation of neurotransmission, deficiencies of neurotrophic factors, or immune dysregulation. New studies on the expression of noncoding RNA in WD are beginning to shed light on potential molecular pathways involved in psychiatric symptomatology. However, current evidence is still insufficient to definitively establish the cause of psychiatric symptoms in WD. It is possible that the etiology of psychiatric symptoms varies among individuals, with multiple biological and psychological mechanisms contributing to them simultaneously. Future studies with larger samples and comprehensive analyses are necessary to elucidate the mechanisms underlying the psychiatric manifestations of WD and to optimize diagnostics and therapeutic approaches.

## 1. Introduction

Wilson’s disease (WD) is an autosomal recessive disorder of copper metabolism [1]. The genetic defect in WD affects the *ATP7B* gene, which encodes the ATP7B transmembrane protein, which is essential for maintaining normal copper homeostasis in the body [2,3]. It is primarily expressed in the liver and acts by incorporating copper into ceruloplasmin (Cp), the major copper transport protein in the blood [2,3]. In conditions of excess copper, ATP7B transports it to bile for excretion. Mutations in *ATP7B* lead to impaired ATP7B function, resulting in copper accumulation in hepatocytes leading to their damage. The toxic “free”—unbound to Cp—copper released from hepatocytes then accumulates in various organs, contributing to their damage and clinical manifestations of WD, including hepatic, neurological, hematological, renal, musculoskeletal, ophthalmological, psychiatric, and other symptoms [4]. The mechanisms of tissue damage in WD include direct toxicity of copper to cells, increased oxidative stress, neurotransmitter disorders (synthesized with copper as a cofactor), cuproptosis, ferroptosis, autophagy, mitophagy, immune dysregulation, and are still not fully understood [5].

While most of the clinical manifestations of WD can be explained by organic or cellular changes (hepatic—hepatocyte necrosis, liver cirrhosis; neurological—copper accumulation in the brain, demyelination due to copper accumulation; hematological—direct copper toxicity to erythrocytes or bone marrow; ophthalmological—copper accumulation in the cornea or lens), there are difficulties in explaining the psychiatric manifestations of WD since there are not direct correlations between the location of brain damage and psychiatric manifestations of WD [6]. In the course of WD, symptoms occur that are difficult to explain solely by brain damage caused by copper (e.g., mood disorders, psychotic disorders, personality or cognitive disorders, sleep disorders, etc. [7,8,9,10,11,12,13,14,15,16,17,18,19,20,21,22]). There are several cases of WD described in the literature in which the patient had clinical psychiatric symptoms but did not have pathological brain changes documenting copper accumulation in neuroimaging studies, in contrast to the neurological presentation of WD, in which brain changes are observed in 100% of patients [23]. This raises the question of whether the psychiatric symptoms observed in WD are caused by *ATP7B* mutations and are a consequence of copper accumulation in the brain, or whether they are simply a coincidence or a reaction to the diagnosis of a genetic disorder.

## 2. Methods

The aim of our narrative review was to briefly characterize the psychiatric manifestations of WD (different symptoms, age of onset, prevalence, and possible treatments) and then discuss possible causes of their occurrence. To determine what psychiatric symptoms were described in patients with WD and how frequently they occurred, we conducted a focused literature review of articles available in English and published up to 1 October 2024. Search filters were used to target human studies published in English. A search for relevant articles was conducted in PubMed/Medline, Science Direct, Scopus, Willy Online Library, and Google Scholar databases combining free-text and MeSH terms using a broad range of synonyms and related terms, which were previously applied by Mura et al. [23], such as the following: (“mental disorders”[MeSH Terms] OR (“mental”[All Fields] AND “disorders”[All Fields]) OR “mental disorders”[All Fields] OR (“psychiatric”[All Fields] AND “disorders”[All Fields]) OR “psychiatric disorders”[All Fields]) OR (“depressive disorder, major”[MeSH Terms] OR (“depressive”[All Fields] AND “disorder”[All Fields] AND “major”[All Fields]) OR “major depressive disorder”[All Fields] OR (“major”[All Fields] AND “depressive”[All Fields] AND “disorder”[All Fields]) OR “major depressive disorder” [All Fields] OR “depressive disorder”[MeSH Terms] OR (“depressive”[All Fields] AND “disorder”[All Fields]) OR “depressive disorder”[All Fields] OR (“major”[All Fields] AND “depressive”[All Fields] AND “disorder”[All Fields])) OR (“bipolar disorder”[MeSH Terms] OR (“bipolar”[All Fields] AND “disorder”[All Fields]) OR “bipolar disorder”[All Fields]) OR (“mania” [All Fields]) OR (“psychotic disorders”[MeSH Terms] OR (“psychotic”[All Fields] AND “disorders”[All Fields]) OR “psychotic disorders”[All Fields] OR (“psychotic”[All Fields] AND “disorder”[All Fields]) OR “psychotic disorder”[All Fields]) OR (“schizophrenia” [MeSH Terms] OR “schizophrenia”[All Fields]) OR (“anxiety disorders”[MeSH Terms] OR (“anxiety”[All Fields] AND “disorders”[All Fields]) OR “anxiety disorders”[All Fields]) AND (“hepatolenticular degeneration”[MeSH Terms] OR (“hepatolenticular”[All Fields] AND “degeneration”[All Fields]) OR “hepatolenticular degeneration”[All Fields] OR (“wilson’s” [All Fields] AND “disease”[All Fields]) OR “Wilson’s disease”[All Fields]).

To assess the type and frequency of psychiatric symptoms in WD, we thoroughly searched and analyzed all relevant references. Initially, titles and abstracts of articles (4580 articles) retrieved according to the search terms were independently searched by two authors (AA and TL) and duplicate records were removed. After evaluation and corrections, overlapping studies were excluded as well as articles not related to the research topic. Then, the full text of initially eligible articles (200) was searched. Finally, all identified studies were independently analyzed by two authors (AA and TL) to confirm the inclusion criteria and, finally, 161 articles were selected for review. All authors separately reviewed articles that might be useful according to the following guideline: only studies written in English with any research design focusing on psychiatric comorbidity in WD. Only studies published in peer-reviewed journals were analyzed, which allowed us to assume that the methodology was assessed by at least two reviewers and therefore the study results can be considered reliable.

Since we did not conduct a systematic review but only a narrative review, we did not use risk of bias scales such as (Newcastle–Ottawa scale, Pedro scale, etc.) and all articles meeting the inclusion criteria were analyzed; additionally, reference lists were reviewed, from which additional articles meeting our criteria that were not identified in the previous searches were extracted—these articles were also analyzed and finally discussed. We did not include papers, abstracts, or articles from conferences, symposiums, or posters that have not been published. The study period was from 1985 to 2024. The name of the first author and the year the study was published along with the sample size, study design, assessment, and results of the chosen articles were entered into a spreadsheet. Reviewers worked together to resolve disagreements by talking about them. The search took place between August 2024 and October 2024. The selected articles (161) were then carefully re-read by all authors, analyzed, and summarized. The results of these studies were finally summarized in a narrative form describing the analyzed problems.

## 3. Results

### 3.1. WD—Clinical Symptoms

As mentioned above, the clinical manifestations of WD are caused by pathological accumulation of copper in various tissues and organs, which leads to their damage and clinical symptoms. According to the pathogenesis of WD, the primary clinical manifestations of WD include hepatic and/or neuropsychiatric symptoms. The disease usually begins with hepatic symptoms (up to 60% of patients with WD have hepatic symptoms at diagnosis and during the course of the disease almost 100% suffer from hepatic symptoms). Hepatic manifestations include initially increased serum aminotransferase levels (clinically asymptomatic), further acute or chronic hepatitis, steatosis and finally (in untreated cases) cirrhosis (compensated or decompensated). Thus, hepatic manifestations can be extremely variable from almost only abnormal laboratory results to cirrhosis or acute liver failure [1,17,18]. Because WD begins in the liver, clinical signs of liver damage usually appear earliest, in the first decade of life. The youngest reported patient with WD diagnosed with elevated aminotransferase levels was a 13-month-old child, while the youngest patient diagnosed with cirrhosis was a 3-year-old.

During the disease, copper accumulates in other organs (brain, cornea, kidney, etc.), which leads to further clinical manifestations of WD. The neurological picture belongs to the second common form of WD and includes mainly movement disorders (tremor, dystonia, bradykinesia, chorea) associated with drooling, dysphagia, and difficulties with gait and posture [17]. These symptoms usually appear between the third and fourth decade of life, but the youngest described patient with neurological symptoms of WD was 6 years old and the oldest was 72 years old.

Importantly, almost all patients with WD with neurological symptoms have typical changes in magnetic resonance imaging (MRI) of the brain—symmetric hyperintense or mixed intensity changes in T2-weighted images located in the basal ganglia (putamen, globus pallidus, thalamus, midbrain); in advanced cases these changes are visualized as hypointense (irreversible); in the case of hepatic WD, the changes are visualized less frequently (in up to 40% of patients, mainly in the globus pallidus) [6,17].

The third most common manifestation of WD is psychiatric and it will be discussed separately (as the main topic of this article) [6,7,8,9,10,11,12,13,14,15,16,17,18,19,20,21,22,23]. Ophthalmological manifestations of WD, as pathognomonic for WD, include the Kayser–Fleischer ring (K–F ring) as well as sunflower cataracts (rarely occurring in WD). The K–F ring is present in almost 100% of patients with neurological WD, in 40% of patients with hepatic WD, and in 20% of asymptomatic patients and is included in the Leipzig scale, the diagnostic algorithm for WD [17]. Much rarer presentations of WD include renal abnormalities including aminoaciduria, nephrolithiasis, hypercalciuria, and nephrocalcinosis; cardiac abnormalities—cardiomyopathy; osteoskeletal pathology—chondrocalcinosis and osteoarthritis; and infertility or recurrent miscarriages [6,17].

### 3.2. Psychiatric Symptoms in WD

Psychiatric symptoms of WD are, in addition to hepatic and neurological symptoms, among the most common clinical symptoms of WD [6,7,8,9,10,11,12,13,14,15,16,17,18,19,20,21,22,23,24,25,26,27,28,29,30,31,32,33,34,35,36,37,38]. An analysis of a multicenter international WD registry found that approximately 37% of patients had a lifetime history of major depressive disorder and 6% had had an active episode of major depression [19]. There are anecdotal reports of mania secondary to WD [39], in which electroconvulsive therapy may be effective [40]. A recent review found that 20% of patients had seen a psychiatrist before being diagnosed with WD [41].

The prevalence of various mental disorders in patients with WD ranges from 4 to 47% for major depressive disorder and from 1.4 to 11.3% for psychosis [28].

The co-occurrence of neurological symptoms may complicate psychiatric examination and contribute to both underdiagnosis (due to the tendency to provide a reactive explanation) and overdiagnosis of psychiatric disorders (when neurological symptoms co-occur with symptoms such as apathy, psychomotor retardation, and sleep disorders) [42].

Psychiatric symptoms of WD are among the greatest challenges in the treatment of WD [9,28,43,44,45]. First, early diagnosis of WD in patients whose disease manifests for the first time in the form of psychiatric symptoms is difficult [20,27,46,47]. Second, there are no guidelines for the treatment of psychiatric symptoms in WD that take into account the specificity of this disease [48]. The presence of psychiatric symptoms in the absence of neurological or hepatic symptoms is one of the most confusing features of the onset of WD.

The first data on the occurrence of mental disorders in WD were published by S.A. Wilson, who, in 1912, describing 12 cases of hepatolenticular syndrome (hereinafter referred to as Wilson’s disease), found in 66% of patients (8/12) so-called “psychic” symptoms, which included psychosis (delusions, hallucinations, etc.), cognitive disorders (described as “apathy, slowness of thought processes, simplicity of mind, loss of memory and mental powers”, etc.), behavioral disorders (“restless, unable to concentrate on anything ..., easily provoked to laughter, childish”, etc.), and mood disorders (mainly depression) [49]. Wilson described these symptoms as highly variable in degree and type. In analyzing these symptoms, Wilson divided them into four main groups: (1) narrowing of the mental horizon (cognitive disorders—but not dementia, he did not find aphasia, apraxia, agnosia, or disorientation in space and time in any of the described patients with WD); (2) ease, docility, and childishness (behavioral disorders); and (3) psychosis. In addition, he described a fourth group of symptoms probably referring to the involvement of other organs in WD with predominant sickness and bulimia (with possible involvement of digestive disorders) [11,17,33]. However, the high frequency (66%) of so-called “psychological” symptoms in patients with WD and their great heterogeneity made Wilson uncertain whether their occurrence in WD was a coincidence or whether they were caused by WD. And, such a dilemma, whether psychiatric symptoms are caused by WD (copper toxicity or hepatic encephalopathy (HE)) or whether they are simply a coincidence, remains to this day. The high frequency of these symptoms in patients with WD makes them very important in the diagnosis of WD as well as in the treatment [28].

Current epidemiological data suggest that up to 30% of patients with WD begin the disease with psychiatric symptoms and, during the course of the disease, almost 100% of patients with WD present some kind of psychiatric symptoms of varying severity (mostly mild) [12,14,19,29,30,31,32,33,34,35,36,37,38].

In cases with initial hepatic symptoms, the median time to diagnose WD is 6 months; in cases with initial neurological symptoms, it is 18 months. Unfortunately, in patients with psychiatric symptoms, the median time to diagnose WD is 26 months and delays of up to 12 years have been reported [17,48].

Some clinical observations indicate that acute psychiatric symptoms may appear immediately after initiation of treatment with anticopper drugs or within the first few months of treatment, paradoxically even when the patient’s neurological condition is improving.

Psychiatric symptoms of WD can have a negative impact on the recovery and even survival of patients [7,8,9,17]. This may be due to delays in making the correct diagnosis, the negative impact of psychiatric disorders on compliance with treatment, and the adverse effects of psychiatric medications (e.g., neurological deterioration, malignant neuroleptic syndrome, drug-induced liver failure) [50,51,52,53,54].

### 3.3. Current Classification of Psychiatric Symptoms in WD

A wide spectrum of psychiatric, psychological, and psychosomatic symptoms has been described in patients with WD. They include mood disorders (mania, depression, suicide), cognitive disorders (from poor school performance through mental retardation and selected deficits in attention, learning, memory, etc.), behavioral and personality disorders (aggression, irritability, antisocial behavior, emotional lability, etc.), psychotic disorders (states similar to schizophrenia, delusions, hallucinations, etc.), and others such as anxiety disorders, anorexia-bulimia, sleep disorders, confusional states, etc. Based on their frequency of occurrence as well as clinical symptoms, Carta et al., in 2012 [11] proposed dividing them into five groups: (1) cognitive disorders; (2) personality and behavioral disorders; (3) mood disorders; (4) psychosis and (5) other mental disorders. To this day, this classification is widely used by doctors involved in the diagnosis and treatment of WD as well as by scientists.

### 3.4. Types, Prevalence, and Possible Treatment Options for Psychiatric Symptoms in WD

As mentioned above, psychiatric symptoms in WD can be divided into five groups, which are presented in detail below along with data on their prevalence in WD and possible pharmacological and nonpharmacological treatment [17,28].

#### 3.4.1. Cognitive Disturbances

Cognitive disturbances are reported in 25% of WD patients and were first described by S.A. Wilson [49]. As mentioned earlier, he described a narrowing of the mental horizon, not a true dementia (without cortical cognitive deficits such as apraxia, agnosia, aphasia, etc.). In the 1980s, these cognitive impairments in WD began to be defined as subcortical dementia or even reversible dementia (due to the reversibility of some deficits associated with copper toxicity or hepatic encephalopathy (HE)) [11,19,27]. Currently, based on several neuropsychological studies, there is a detailed description of the cognitive impairments that can be observed in WD. They usually include selected deficits in attention, visuospatial perception, reasoning, verbal and abstract reasoning, learning, and memory. Patients with WD may also have slow processing speed as well as mild impairment in all cognitive domains, which correlates with the global severity of brain magnetic resonance imaging (MRI) [27]. Consciousness disturbances may only occur in severe HE and in the end-stage of the disease. Importantly, improving liver function tests as well as anticopper treatment of WD may improve cognitive impairment (HE treatment and anticopper treatment reduce the direct toxic effects of copper on neurons) [27,43,44,45]. There is no specific treatment for cognitive impairment in WD apart from appropriate treatment of WD and HE, which should improve deficits. Neurophysiological rehabilitation may be additionally helpful but as an adjunctive method to the causal treatment of WD and liver failure.

#### 3.4.2. Behavioral and Personality Disorders

Behavioral and personality disorders are among the most common and most difficult-to-treat psychiatric symptoms of WD, observed in approximately 46–71% of patients during the course of the disease [11,17,27]. Most often, they include irritability, aggression, and/or antisocial behavior. Very often, these symptoms correlate with neurological hyperkinetic symptoms (dyskinesia), dysarthria, and changes in the MRI image of the brain located in the putamen and globus pallidus. All these symptoms can lead to social problems (criminal activity, unemployment, divorce, etc.) and also create difficulties in the treatment of WD (aggression, mainly verbal toward medical personnel, nonadherence to anticopper therapy or other medications). There are no specific recommendations for the treatment of behavioral and personality disorders in WD. All case reports are based on general psychiatric recommendations, depend on the severity of the disorder (impact on quality of life) and include behavioral therapy, selective serotonin reuptake inhibitors (SSRIs) such as citalopram, escitalopram, sertraline, antiepileptic drugs (carbamazepine, gabapentin, lamotrigine, pregabalin), short-term benzodiazepines (BZDs) such as lorazepam, oxazepam, te-mazepam or beta-blockers (propranolol). In case of severe or refractory psychiatric symptoms (aggression, etc.), atypical neuroleptics (quetiapine or tiapride) should be considered (with caution due to the possibility of exacerbation of neurological symptoms) [27].

#### 3.4.3. Mood Disorders

Mood disorders (in addition to behavioral and personality disorders) appear to be the second-most common psychiatric symptom of WD. Various studies have reported the occurrence of depression in up to 60% (30–60%) of patients, with a high rate of suicide attempts (4–16%) and bipolar disorder (BD) reported in 14–18% of patients [11,12,14,17,19,27,29,30,31,32,33,34,35,36,37,38]. There is no clear answer as to why patients with WD have such high rates of mood disorders but several possible mechanisms have been considered. First, they may represent a reactive response resulting from a chronic medical condition, often associated with a neurological deficit that reduces quality of life due to physical disability and stigmatization, and second, from symptoms of liver failure leading to chronic fatigue and exhaustion. Possible biological and cellular mechanisms of mood disorders related to copper metabolism as well as neurotransmitter abnormalities are discussed in the following sections. The treatment of mood disorders in WD includes all available antidepressants SSRIs, tricyclic antidepressants (TCAs), serotonin–norepinephrine reuptake inhibitors (SNRIs), serotonin antagonists and reuptake inhibitors (SARIs), lithium salts, antiepileptic drugs (carbamazepine, oxcarbazepine, lamotrigine, gabapentin, etc.), atypical neuroleptics (clozapine or quetiapine, as in psychosis), and electroconvulsive therapy, which is used to treat mood disorders in WD. As described earlier, the treatment should be carried out carefully, taking into account the possibility of drug-induced neurological deterioration or liver damage in order to minimize such risks [27].

#### 3.4.4. Psychosis

Psychosis, as a clinical symptom in WD, was first described by Wilson [49] (2/12 patients) but, currently, based on available analyses, its incidence seems to be similar to that in the general population (the expected lifetime risk of psychosis is 7.8%, changing over the lifespan, at around 5–7% in adulthood and a much higher incidence in childhood (17%)) [12,14,19,29,30,31,32,33,34,35,36,37,38]. In patients with neurological symptoms, the incidence of psychosis is slightly higher than in the general population—up to 8%. WD does not have specific psychotic symptoms, so the presence of psychotic episodes (PE) in patients often results in a diagnosis of schizophrenia or schizoaffective or delusional disorders. Since almost 3% of first episodes of psychosis occur due to “organic” causes, this must be taken into account in the differential diagnosis in this group of patients. Pharmacological treatment of patients with psychosis of WD is very difficult and requires special caution due to the high risk of neurological deterioration (in the case of neuroleptics) and/or the additional risk of liver damage associated with the drug. Neuroleptics with a relatively low risk of extrapyramidal symptoms, not metabolized in the liver, such as clozapine or quetiapine, are recommended. In such cases, additional precautions are necessary in patients with WD because clozapine can cause leukopenia (patients with liver cirrhosis or taking DPA often initially have leukopenia), which can lead to severe clinically significant leukopenia. Treatment with neuroleptics in patients with WD may additionally lead to epileptic seizures because they lower the seizure threshold and patients with WD have an initially increased risk of epilepsy (6–8%) [27].

#### 3.4.5. Other Psychiatric Disorders in WD

There are several other psychiatric symptoms that may occur in WD or even be the first clinical manifestation of WD. These include sleep disorders, anxiety, substance abuse, catatonia, bulimia and anorexia, etc. For most of these symptoms, their prevalence in WD has not been estimated but sleep disorders with poor sleep quality and efficiency, with increased wakefulness, have been reported in WD (especially in patients with neurological WD) [12,14,19,27,28,29,30,31,32,33,34,35,36,37,38,55,56,57,58]. In summary, clinical symptoms and psychiatric syndromes in WD do not have unique/classic features that occur only in WD. Due to the high prevalence of these symptoms and their significance (quality of life, suicide, compliance with WD treatment, possible drug-induced neurological and hepatic deterioration, delay in WD diagnosis, etc.), physicians involved in the treatment of WD patients should have adequate knowledge about their diagnosis and treatment. The prevalence of individual psychiatric symptoms in WD in the publications of different authors is presented in Table 1.

### 3.5. Possible Pathomechanisms of Psychiatric Symptoms in WD

The mechanisms responsible for the occurrence of psychiatric symptoms of WD are not fully understood. It has been hypothesized that psychiatric symptoms of WD may be a consequence of the disease, the symptoms and diagnosis of which can be a serious stressor, often leading to significant changes in life, difficulties in normal functioning, and may also lead to a deterioration of social and material status [59]. Many patients with WD experience various forms of coexisting disorders, including anxiety, depression, worries, insomnia, and tension but also irritability, anger, and behavioral disorders [11]. Thus, the relatively high prevalence of depression in WD was explained by the severity of functional impairment. However, depression is more common in WD than in patients with other chronic diseases characterized by a similar level of disability such as rheumatoid arthritis [60]. On the other hand, the prevalence of depression in WD is similar to the prevalence of depression in other basal ganglia (BG) disorders [42].

Thus, it is highly probable that the cause of the occurrence of depression as well as other psychiatric syndromes in WD may be damage to the BG—this structure can generate various psychiatric syndromes [10,61]. As in the CNS, WD mainly affects the BG [61]—it may be hypothesized that the pathogenesis of neuropsychiatric symptoms may be similar to those seen in other neurodegenerative disorders that involve the BG (e.g., Parkinson’s disease [PD] or Huntington’s disease [HD]) [28].

The role of brain damage in the pathogenesis of psychiatric symptoms in WD is also indicated by observations in patients with cognitive deficits (low processing speed and mild impairment in all cognitive domains, especially in working memory, attention, and abstract thinking), which—according to the research results—may be related to changes in the cortico-striatal pathways and correlate with the global intensity of MRI abnormalities [52,62,63,64,65].

Some authors suggest that there may be a common mechanism for some of the behavioral changes, mood disturbances, and anxiety disorders observed in patients with BG disorders [10,42,61,66] and the involvement of the same neurotransmitter systems (e.g., dopaminergic, noradrenergic, serotonergic) in different disorders may lead to similar recommendations for pharmacological treatment [20,21].

Additionally, in the case of WD, liver involvement should be taken into account when considering the causes of psychiatric symptoms. Ferenci et al. [67] suggested that “minor” psychopathological symptoms (such as apathy, irritability, and emotional lability) may be caused by mild hepatic encephalopathy—this is indicated by the observations of the occurrence of liver cirrhosis in a large percentage of patients with neuropsychiatric symptoms of WD (34% of patients had liver cirrhosis) [68,69]. Thus, liver involvement should also be considered when planning the treatment of neuropsychiatric symptoms with drugs that are metabolized in the liver.

In the CNS, copper is one of the important trace elements involved in brain synaptic transmission (affects N-methyl-D-aspartate (NMDA), gamma-aminobutyric acid-A (GABA-A), voltage-gated calcium channels (VGCC)), glial apoptosis, etc., the disturbances of which are postulated to lead to several psychiatric disorders (mood disorders, cognitive deficits, psychoses, addictions, etc.) [41,70,71,72,73,74,75,76,77,78,79,80,81,82,83]. There are also several other proposals regarding possible mechanisms responsible for the development of psychiatric symptoms in WD, which include: (1) direct and indirect copper toxicity [41,78,79,80,81]; (2) disturbances in the biosynthesis and action of neurotransmitters and neuropeptide metabolism secondary to dysfunctional copper metabolism (dopamine, noradrenaline, serotonin, glutamate, GABA, etc.) [82,83,84,85]; (3) abnormalities in neurotrophic factors (e.g., brain-derived neurotrophic factor; BDNF) [86,87,88,89]; (4) oxidative stress [90,91]; (5) mitochondrial damage, mitophagy, cuproptosis, ferroptosis; (6) genetics—*ATP7B* mutations [58,84] and others that have been described below.

#### 3.5.1. Copper Toxicity

It has been shown that even in healthy conditions, copper is abundantly present in the brain, especially in the basal ganglia, cerebellum, hippocampus, and cell bodies of the pyramidal tracts. This metal is required as a cofactor for many enzymes important for various brain functions. Even minor disturbances in copper metabolism leading to its excess or deficiency can affect various brain functions, leading to neuropsychiatric symptoms.

Under normal conditions, copper is transported in serum to various tissues, mainly in the form bound to Cp (up to 90%; it is a safe, nontoxic copper pool). In the case of WD, most of the serum copper is present in the form unbound to Cp—this is the so-called “free copper” (non-ceruloplasmin-bound copper: NCC) bound to albumin, alpha-macroglobulin, small peptides, and amino acids, which can accumulate in various tissues (including the brain and neurons) leading to oxidative stress (redox cycling reactions) and indirectly affecting sensitive tissues (including neurons), which can cause neurological and psychological symptoms of WD [17].

The possible involvement of copper in the pathogenesis of mental disorders in WD is indirectly confirmed by the results of studies on copper concentration in patients with various mental disorders.

In general, psychiatric disorders constitute a heterogeneous group, mostly with multifactorial etiology. The importance of trace element disturbances, especially copper, as part of their etiology has been widely studied so far with diverse and interesting results [41,70,71,72,73,74,75,76,77,78,79,80,81,82,83]. Squitti et al. analyzed copper metabolism in 109 patients with mood spectrum disorders (MSD) and schizophrenia spectrum disorders (SSD) and found that women with MSD had higher levels of total copper as well as NCC. Based on laboratory and experimental data, it is suggested that only “free copper” (NCC) enters the brain, which can accumulate in the brain and lead to the development of neuropsychiatric symptoms. Therefore, it seems that the pathogenesis of MSD or SSD, associated with the accumulation of NCC, may be similar to the pathogenesis of psychiatric symptoms in WD. When discussing their observations, the authors emphasized the possibility of copper’s influence on estrogen metabolism. They suggested that copper acting as a “metalloestrogen” by activating estrogen receptor alpha affects enzymes regulating estradiol biosynthesis, which may lead to the occurrence of MSD [58]. Other studies evaluating the role of copper in depression conducted by Styczen et al. [74] did not confirm the involvement of copper in the pathogenesis of mood disorders but the authors only examined total copper, which could have influenced the results. However, other reports documented higher serum copper levels in patients with depression [84]. Therefore, due to the conflicting results, the hypothesis regarding the involvement of copper in the pathogenesis of mood disorders should be verified in further studies, including new biomarkers of copper metabolism, such as labile copper, direct NCC (dNCC) or exchangeable copper (CuEXC). Squitti et al. investigated the role of copper in cognitive impairment (Alzheimer’s disease; AD). The authors analyzed 28 AD patients and 29 healthy age-matched controls, analyzing serum Cp concentrations and mean and percentage of “free copper”. The authors found that AD patients had an increased percentage of “free copper” (not bound to Cp) compared to the control group. Absolute “free copper” was higher in the AD group but without statistical significance. The involvement of copper in the pathogenesis of AD has been studied in both animals and humans. Initial studies have shown that adding 0.12 ppm copper to drinking water in mice increased the formation of amyloid plaques as well as damaged lipoprotein-receptor-related protein (LRP), which is involved in the removal of amyloid from the brain. Furthermore, Squitti et al. conducted a meta-analysis of data on copper concentrations in the serum, plasma, and brain of AD patients, finding decreased copper concentrations in the brains of AD patients with increased total copper and NCC concentrations in the serum of AD patients, supporting the copper hypothesis of AD [88]. These observational and experimental studies were further supported by studies in transgenic mice treated with the anticopper preparations clioquinol and tetrathiomolybdate, which reduced brain amyloid pathology. These observations may indicate a pathway by which copper may contribute to cognitive impairment by stimulating amyloid accumulation in the brain. However, we should be cautious about these observations, as cognitive decline in WD patients is not associated with amyloid pathology. Exploring the hypothesis of the involvement of trace elements, including copper, in the pathogenesis and progression of psychiatric disorders, in 2024, Chinese authors conducted a systematic review and meta-analysis analyzing changes in ion levels in the blood and cerebrospinal fluid (CSF) in patients with depression [89]. In an analysis combining data from 75 articles, they showed increased total serum copper in patients with depression and decreased copper levels after SSRI treatment. These results suggest that copper is involved in the pathogenesis of psychiatric disorders, especially mood disorders; unfortunately, the authors did not distinguish between total and “free” copper in their analysis.

Analyzing different ions in patients with depression, the authors suggested that elevated serum copper levels may additionally induce inflammatory processes by reducing anti-inflammatory cytokines (interleukin-4 and 10), increasing proinflammatory cytokines (interleukin-1beta, 2, 6, and 8 and tumor necrosis factor-alpha (TNF-alpha)), which are also considered in the inflammatory hypothesis of psychotic and mood disorders. An explanation for such an effect may be the increased permeability of the brain–blood barrier (BBB), resulting in a greater accumulation of copper in the brain due to peripheral inflammation induced by increased serum copper levels [89,90,91].

#### 3.5.2. Oxidative Stress

One of the hypotheses regarding the causes of the occurrence of mental disorders in WD assumes that copper determines brain damage in WD through oxidative stress. Increased values of oxidative stress markers, mainly related to lipid peroxidation, have been documented in patients with bipolar disorder (BD) [92]. Evidence has been accumulated for a specific correlation between WD and BD, which may have a similar pathogenesis due to oxidative damage to the brain and consequently neurodegeneration caused by the accumulation of trace metals [33]. Increased oxidative stress in the brain causes deleterious effects on signaling, structural plasticity, and cellular resilience, mainly by inducing peroxidation of membrane lipids, proteins, and DNA [93]. It has been hypothesized that these pathological processes occur in critical brain circuits that are important for affective functioning, emotions, motor behavior, and pleasure [94,95].

As copper is a redox-active metal, and tissue copper overload is associated with reactive oxygen species (ROS) formation via the Fenton reaction [96], it may be hypothesized that oxidative stress may represent the main pathophysiological mechanism of CNS damage and psychiatric manifestations of WD.

The brain is particularly vulnerable to oxidative damage due to its comparatively high oxygen utilization and, hence, generation of free radical by-products, its modest antioxidant defenses, its lipid-rich constitution that provides ready substrates for oxidation, the reducing potential of central neurotransmitters, and the presence of redox-catalytic metals such as iron and copper [5,67,97]. Increased neuronal oxidative stress induces deleterious effects on signal transduction, structural plasticity, and cellular resilience, mostly by inducing lipid peroxidation in membranes, proteins, and DNA [98]. It has been hypothesized that these pathological processes occur in critical brain circuits that regulate affective functioning, emotions, motoric behavior, and pleasure [94,95].

Over the last decade, a great deal of information has appeared in the psychiatric literature on the mechanisms of oxidative stress. There was published evidence of increased oxidative stress in various psychiatric disorders, including autistic disorder, amphetamine-related disorders, hallucinogen-related disorders, nicotine-related disorders, sexual dysfunctions, eating disorders, sleep disorders, opioid-related disorders, schizophrenia and other psychotic disorders, mood disorders, anxiety disorders, behavioral disorders, such as aggressive behavior, and depression, as well as to deterioration of short-term spatial memory and mental retardation [99,100]. Psychotic disorders, including schizophrenia, are therefore characterized by the depletion of compensatory mechanisms related to the action of antioxidants. This leads to a shift in the oxidative–antioxidant balance in favor of prooxidant factors, increasing oxidative damage to macromolecules such as proteins, lipids, and nucleic acids. Accumulation in the brain of oxidative modifications of proteins or lipids, which can cause serious neuronal damage, is a direct cause of psychiatric symptoms in schizophrenia [101,102,103].

In WD, psychiatric symptoms develop more than 5 years later than the hepatic ones. This may be partially explained by the fact that the brain’s sensitivity to oxygen increases with age, as a natural consequence of the brain aging is a declining ability of the antioxidant system to prevent oxidative damage resulting from non-detoxified reactive oxygen species (ROS) [104,105].

#### 3.5.3. Mitochondrial Dysfunction, Cuproptosis, Ferroptosis

The proper functioning of neurons is largely determined by the undisturbed functioning of the mitochondrial electron transport system in the respiratory chain.

It generates high amounts of ATP energy, which is needed to maintain cellular homeostasis and proper functions related to electrical activity or synaptic transmission. For this reason, even minor disturbances in mitochondrial function can impair neuronal function and contribute to the development of neuropsychiatric disorders, with subsequent extracerebral consequences and activation of alternative ATP synthesis pathways [102,106,107,108].

For example, in schizophrenia, bioenergetic anomalies have been observed in the early stages of psychosis. This proves that in patients, energy in the form of ATP is stored in the form of phosphocreatine in smaller amounts as opposed to NADH (reduced form of nicotinamide adenine dinucleotide), which is accumulated in these patients. The increase in NADH in people suffering from mental disorders indicates a redirection of mitochondrial function from aerobic respiration to glycolysis in response to decreasing efficiency of mitochondrial ATP levels [108].

It has been shown that patients with primary mitochondrial diseases caused by gene mutations in nuclear and mitochondrial DNA are at a multiple increased risk of developing BD [109,110].

Many studies have proven that mitochondrial metabolism is damaged/disturbed in WD. This may be a consequence of two mechanisms important for cell death in WD: cuproptosis and ferroptosis. It has been shown that in WD, not only the metabolism of copper but also iron is disturbed because the metabolic pathways of these two elements are linked at several stages. Many studies have shown that these two elements accumulate in the tissues of patients with BD [5,111,112,113]. Both metals are redox active and participate in the Fenton and Haber–Weiss reactions, which lead to the formation of free oxygen radicals but also many other disorders leading to cell death through recently described mechanisms, which have been called cuproptosis and ferroptosis.

In both types of cell death, mitochondria and mitochondrial metabolism play an important role. It has been proven that copper cytotoxicity impairs mitochondrial integrity by accumulating lipoylated proteins and causing the loss of Fe–S cluster proteins, which leads to cuproptosis [114,115]. The main morphological features of cuproptosis are mitochondrial degeneration, membrane damage, endoplasmic reticulum damage, and chromatin damage like apoptosis. Ferroptosis is also characterized by specific morphological changes in mitochondria, including decreased volume, increased membrane permeability, disruption of the outer mitochondrial membrane, and loss of mitochondrial cristae. In addition to morphological changes, ferroptosis affects mitochondrial function, leading to decreased ATP synthesis and DNA damage. Ferroptosis involves morphological changes in mitochondria such as shrinkage and increased membrane and mitochondrial fragmentation, which are not observed in cuproptosis [5,116,117].

Therefore, it can be assumed that mitochondrial disorders may be important in the pathogenesis of mental disorders in WD.

#### 3.5.4. Changes in Neurotransmitters

As mentioned previously, copper as a cofactor for many enzymes is involved in the biosynthesis of many neurotransmitters, e.g., the conversion of dopamine to norepinephrine is copper dependent (dopamine-beta-monooxygenase; DBH) and modulates glutaminergic synaptic transmission, GABA-A receptors, NMDA receptors, and others. Further, copper affects monoamine oxygenase-A and B (MAO-A and B), which is involved in the reuptake of serotonin, dopamine, and noradrenaline in such a way that affects the metabolism of key neurotransmitters involved in the pathogenesis of mood disturbances and psychosis [41,79,80,81,82,83,84].

Several studies in WD-depressed patients documented serotonin changes, assessing the presynaptic serotonin transporter (SERT) with single-photon emission computed tomography (SPECT) of thalamus-hypothalamus regions and their negative correlations with the Hamilton Depression Scale (HAM-D). Additionally, copper may bind to serotonin, inducing oxidation and structural modification of serotonin leading to serotoninergic deficits and depression [118,119,120,121].

Additionally, it has been proven that copper increase and release is associated with the activation of NMDA receptors and the treatment with NMDA receptor antagonists (memantine) decreases serum copper levels, which could explain links with glutamine activity elevated serum copper level and mood disorders [86,87].

All these mechanisms affecting neurotransmitter biosynthesis and their synaptic reuptake may lead to psychiatric disturbances in WD.

#### 3.5.5. Changes in Neurotrophic Factors

In addition, elevated copper levels may negatively affect the proliferative activity of neurotrophic factors such as BDNF or neuronal growth factor (NGF), which may additionally cause neuropsychiatric symptoms in WD. However, further studies are needed to clarify these effects [87].

#### 3.5.6. Pineal ATP-Ase

Pineal night-specific ATPase (PINA) is a copper-dependent splice variant of *ATP7B* that may be involved in cirrhotic rhythms, sleep, and the pathogenesis of depression, especially in cases of impaired copper metabolism (as in patients with WD). However, there are no studies yet on PINA in WD [24,25].

#### 3.5.7. *ATP7B* Gene Variants

When analyzing the pathogenesis of psychiatric symptoms in WD, there is no clear answer as to whether it is simply a coincidence or a reaction caused by a genetic disease (liver or neurological deficiencies) or the influence of the disease. However, considering the accumulation of copper in the brain with organic changes (located mainly in the basal ganglia but which can occur in all brain structures), the accumulation of manganese in the basal ganglia (in the course of liver disease), and the possibility of clinical or subclinical hepatic encephalopathy (HE) in the course of the disease, the pathogenesis of psychiatric symptoms in WD must be caused by WD. The above-mentioned works documenting the importance of an increased ratio of “free copper” in mood, sleep, and cognitive disorders additionally support the thesis that copper metabolism disorders in WD may lead to the psychiatric manifestation of WD. Looking for genotype–phenotype correlations in patients with WD, there are currently no proven, clear, and repeatable correlations documenting the impact of *ATP7B* variants on clinical symptoms [17].

When analyzing WD with psychiatric symptoms, there are very limited data on genotype–phenotype correlations in WD. There is only one study from 2001 by Portal et al. in which the authors analyzed 25 patients with WD using the Karolinska scales of personality (KSP) and the comprehensive psychopathological rating scale (CPRS) and found that patients homozygous for the Trp779Stop mutation in the *ATP7B* gene had the highest scores on the psychopathy scale compared to conformity and the socialization scale and low scores on the impulsivity, avoidance, and detachment scales. Compound heterozygous variant p.His1069Gln/Arg1319Stop patients with WD had the lowest scores on the psychopathy scale compared to conformity. The authors documented that WD patients with p.Trp779Stop and p.Thr977Met mutations had undetectable Cp levels, so more severe copper metabolism abnormalities, which again emphasized the importance of copper metabolism in psychiatric disorders [58].

However, it should be remembered that in WD, the same genetic variability may be associated with a very diverse clinical course, which applies even to family members or monozygotic twins [122,123,124,125]. For example, out of two sisters carrying the same genotype of the *ATB7B* gene [c.3207C > A/c.3904-2A > G], one showed pronounced neuropsychiatric symptoms (i.e., tremor, lack of motor coordination, and language and cognitive disorders) whereas the other was characterized by liver involvement only [125]. These examples highlight the complexity of interpreting the available data in this field and indicate the possible importance of epigenetic factors in influencing the phenotype, even in a monogenic disease such as WD as it was hypothesized by Czlonkowska, Gromadzka, and Chabik [125].

These examples indicate the complexity of the genotype–phenotype relationship in WD, which also applies to psychiatric symptoms; it can be assumed that epigenetic factors may play an important role in the modulation of the phenotype.

#### 3.5.8. Immunological/Autoimmunological Processes

The research results published in recent years suggest that psychotic disorders may be characterized by the depletion of compensatory mechanisms related to the action of antioxidants, which leads to a shift in the oxidative–antioxidant balance in favor of pro-oxidant factors, increasing oxidative damage to macromolecules: proteins, lipids, and nucleic acids. This may contribute to the initiation of a cascade of proinflammatory events [108,126,127]. In the context of the relationship between oxidative stress and the development of inflammation and psychosis, the concept of mitochondrial DNA release seems important, which, during cellular stress, can induce inflammation associated with neuropsychiatric disorders [128].

According to another concept, inflammation and oxidative stress can cause neurotransmission disorders, leading to psychosis.

Genome-wide association analysis and postmortem studies of patients with psychosis have shown that immune dysregulation may be a fundamental feature of the disease. Inflammatory imbalances—associated with the release of proinflammatory cytokines activating microglial cells—have a synergistic effect with genetic or environmental factors that may affect the occurrence of psychosis-related changes in the brain [129,130].

In the case of mental disorders, including schizophrenia, the role of immunological processes in etiology and course has been demonstrated. In schizophrenia, increased numbers of natural killer (NK) cells, naive B cells, CXCR5 memory cells, and classical monocytes have been demonstrated and decreased numbers of dendritic cells (DCs), HLA-DR+ regulatory T cells (Treg), and CD4+ memory cells have been demonstrated [131].

In the case of first-episode psychosis (FEP), an increased CD4/CD8 ratio has been observed and antipsychotic treatment led to a decrease in this ratio and an increase in CD56 levels [132].

It appears that the key signaling molecules are cytokines, which coordinate the course of innate and adaptive immune processes and affect the peripheral and CNS.

An increase in the level of markers of the state (interleukin 1 beta (IL-1β), 6 (IL-6), and transforming growth factor beta (TGF-β)) was noted during acute psychotic episodes and a return to normal during clinical stabilization. In patients treated chronically, as well as in those in a state of acute psychotic decompensation, an increase in the level of such inflammatory markers such as IL-12, interferon-gamma (INF-γ), tumor necrosis factor (TNF-α) and receptor 2 (sIL-2R) was noted [132]. The inflammatory process in mental disorders may take place not only in the periphery but may also concern the CNS, where activated microglial cells may participate [133]. Activation of microglia has been observed in schizophrenia and other mental illnesses—microglial activation has also been described in Wilson’s disease [23].

It has been shown that microglia can maintain immunological memory in neuropathology, which in turn is associated with an increased response to new systemic inflammation [134].

Evidence suggests that patients with psychotic episodes and diagnosed schizophrenia have higher levels of antibodies than healthy individuals [135]. Serum autoantibodies to the NMDA receptor, a voltage-gated potassium channel, have been demonstrated in individuals with FEP [136,137]. Recent studies suggest that serum antibodies against neuronal cell surface molecules and autoantibodies against the PAGE (prostate-associated gene) group of proteins (including PAGE2B/PAGE2/PAGE5) also appear in this group of patients [138]. In addition, high levels of anti-TRANK1 (thioredoxin peroxidase-related activator of NF-κB and c-Jun N-terminal kinase) IgG were demonstrated in FEP patients, which could probably serve as a biomarker for identifying patients with schizophrenia [139].

In our recent review, we described in detail the inflammatory mechanisms that may play an important role in the pathogenesis of liver damage as well as in the development of CNS pathology in WD [5].

Copper has been shown to accumulate in the striatum of mouse models of WD—this is associated with an increase in the levels of cytokines and chemokines: IL-6, IL-8, IL-10, and TNF-α in this region. It was noted that the intensity of the inflammatory response in different brain regions was dependent on the amount of accumulated copper. Serum IL-6 and IL-8 levels increased with increasing neurological severity (at the same time, GSH and TAC values decreased) [140]. These observations confirm the relationship between oxidative stress, inflammation, and neurodegeneration in WD. In neurological patients, a significantly higher number of Th 1 cells (IL-2, TNF-α, and TNF-β), Th2 cells (IL-13), and Th 17 cells (transforming growth factor-β1 (TGF-β1) and IL-23) was observed compared to the control group (*p* < 0.01). A higher number of Th1 cells (IL-2, TNF-α, and TNF-β), Th2 cells (IL-13), and Th17 cells (TGF-β1, IL-23) was correlated with a greater severity of neurological symptoms [141].

Carrying the *IL1RN* VNTR (variable number of tandem repeats) *2 allele (potentially related to enhanced inflammation) favored the earlier onset of clinical symptoms of WD, especially in patients with the neuropsychiatric form of the disease [142].

These data suggest that increased copper concentration in the CNS is associated with oxidative stress and neuroinflammation, which affects the homeostasis of the microenvironment in the CNS. This may be related not only to the occurrence of neurological symptoms but also to various mental disorders in patients with WD.

In patients with WD, the occurrence of various autoantibodies has also been reported, which we have described in the only review published so far summarizing inflammatory/immune/autoimmune processes in WD. The cause and clinical significance of antinuclear antibodies (ANA) and antineutrophil cytoplasmic antibodies (ANCA) in WD are unclear but may reflect immune system dysregulation. It is possible that autoantibodies may play a role in the pathogenesis of psychiatric disorders in patients with WD.

**Figure 1:**
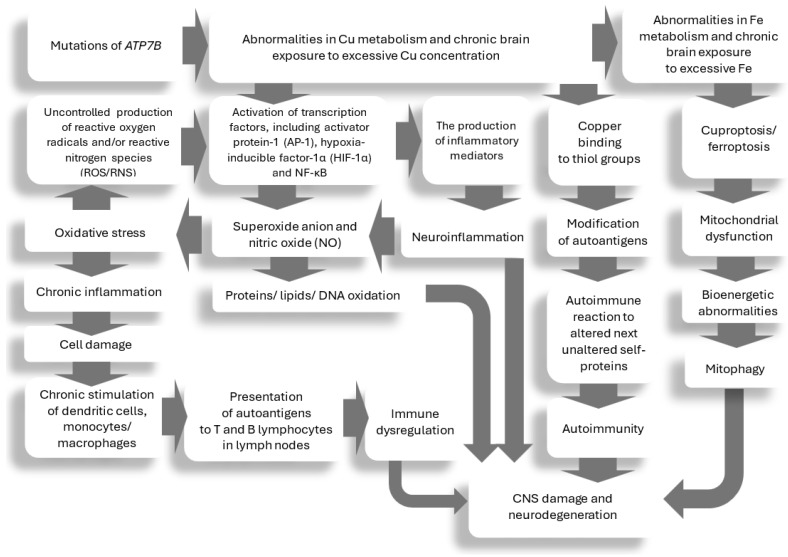
The main mechanisms that may be important in the pathogenesis of psychiatric symptoms of WD.

The main mechanisms that may be important in the pathogenesis of psychiatric symptoms of WD are presented in Figure 1.

### 3.6. Possible Pathogenesis of Psychiatric Symptoms in WD—Additional Conclusions from Experimental Studies

Some light on the mechanisms underlying mental disorders in WD is shed by recently published results of studies conducted using an animal model of WD, called tx mouse. A toxic milk (tx) mouse is an animal model of WD, resulting from an autosomal recessive mutation in the *Atp7b* gene found in the C57BL/6J mouse strain. This mutation occurred spontaneously in the laboratory at the University of Massachusetts (MA, USA). A different recessive mutation in the *Atp7b* gene was identified in the same strain at the Jackson Laboratory (ME, USA). The toxic milk mutation in this strain (txJ) is a point mutation at position 2135 (in exon 8), causing a glycine to aspartic acid substitution (G712D) in the ATPase protein [143]. The txJ mice exhibit copper accumulation in the liver, brain, and other organs leading to phenotypic characteristics similar to those seen in WD [144,145,146].

#### 3.6.1. Mitophagy

Studies on the toxic milk (TX) model have shown that cognitive dysfunction in WD may result from the overactivation of mitochondrial autophagy [147].

Mitophagy is considered to be a selective process of removing damaged mitochondria, which can maintain the balance between the quantity and quality of mitochondria [148,149]. It has been reported that increased mitophagy alleviates mitochondrial dysfunction after neuronal degeneration [150,151]. However, harmful autophagic processes can lead to cell death and impaired mitophagy can protect neurons from brain damage [152].

It is widely believed that failure to properly remove damaged mitochondria or excessive degradation leads to cell death [153,154]. So far, some studies have shown that excess inorganic copper induces oxidative stress and mitophagy, which may lead to mitochondrial dysfunction and then enhance cell apoptosis [155,156,157]. However, the role of mitophagy in WD remained unclear.

Polishchuk et al. have shown that mitochondria in ATP7B-deficient cells were preferentially subjected to autophagy [158]. Therefore, regulation of mitochondria, especially in mitophagy, would be a novel therapeutic approach for WD.

Recent studies indicate that mitophagy is promoted by parkin via specific targeting of mitochondria with loss of membrane potential [159]. The upstream activator of parkin is pink1, which is required both to induce parkin activity and to direct it to these depolarized mitochondria [160,161,162]. In healthy mitochondria, pink1 is continuously imported to the inner membrane, where it is cleaved by PALP (presenilin-related rhomboid protein) [163]. As a result, under normal conditions, pink1 levels remain low and are rarely detectable. However, when mitochondria are severely impaired, their membrane potential drops, halting pink1 transport. The resulting accumulation of pink1 on the outer mitochondrial membrane attracts parkin from the cytosol. Parkin then facilitates the binding of the receptor protein SQSTM1 (ubiquitin-binding adaptor, also known as p62) to mitochondria by ubiquitinating multiple substrates. After the attachment of the phagocytic membrane protein LC3, mitochondria are degraded by lysosomes [164,165]. LC3 exists in two forms: LC3-I (cytoplasmic) and LC3-II (processed) and serves as the main protein marker of mature autophagosomes. Currently, the LC3II/LC3-I ratio is considered a reliable measure to track autophagy and related cell death processes [166].

Using the TX mice model of WD, Jing Zhang et al. [148] demonstrated increased mitophagy in hippocampal tissues; the levels of pink1, parkin, and LC3II (or LC3II/LC3I ratio) were increased in the studied animals, while p62 levels were decreased.

It can be hypothesized that excessive activation of mitophagy in the hippocampus may worsen brain damage and contribute to cognitive dysfunction or other psychiatric symptoms observed in WD. With increased mitophagy, energy metabolism would be impaired, mitochondria and macromolecules would be overused, and this in turn leads to increased neuronal apoptosis [167,168].

#### 3.6.2. Processes/Mechanisms/Biomarkers Identified in Transcriptomic Studies

Some light on the possible pathophysiology of psychiatric disorders in WD is shed by the results of recently published studies on the analysis of changes in the expression of noncoding RNA (ncRNA). ncRNAs are a type of RNA molecule that are not a template for protein synthesis. They constitute about 98–99% of the total RNA in mammalian genomes [169]. There is growing evidence that ncRNAs perform important regulatory functions through interactions with other molecules. For example, ncRNAs bind to one or more target molecules, controlling cells or pathways that affect physiological or disease processes, including the regulation of neurological diseases [170]. Circular RNAs (CircRNAs) are a group of uniform cyclic molecules that are covalently closed and do not have 5′-caps or 3′-polyadenylated tails [171]. They are stable, abundant, and conserved, which gives them unique potential for medical research.

Studies have confirmed that CircRNAs have various important biological functions, such as transcriptional regulation, microRNA (miRNA) adsorption, and gene expression control. CircRNAs are abundant in the mammalian brain, increase during neuronal differentiation, and are abundant in neurosynapses. CircRNAs have been shown to play an important role in the development of neuropsychiatric disorders [172]. The mmu_circ_0001859 and mmu_circ_0000242 genes have been shown to be closely associated with processes such as axon guidance, Wnt signaling, calcium signaling, mitogen-activated protein kinase (MAPK) pathway, focal adhesion, neurotrophin signaling, transforming growth factor-β (TGF-β) pathway, cell cycle, ErbB signaling, notch pathway, p53 signaling, Fc gamma R phagocytosis, and others.

Unlike CircRNAs, long noncoding RNAs (lncRNAs) are less than 200 nucleotides long, with 5′ and 3′ termini as well as polyadenylation tails [173].

Previously, lncRNAs were considered transcripts with no significant biological function [174]. Over the past decade, an increasing number of studies have demonstrated the involvement of lncRNAs in various processes and they are gaining increasing attention as a promising new mechanism of biological regulation [175,176]. Many studies show that lncRNAs have the ability to influence and regulate gene expression by competing for the same genes at the same site. lncRNAs and mRNAs can interact with each other and form a competing endogenous RNA (ceRNA) network [177]. Knowledge of these phenomena may contribute to understanding the mechanisms underlying gene and regulatory networks. ceRNAs may be involved in the regulation of disease-related target genes at both transcriptional and post-transcriptional levels [178,179]. The exact role of most lncRNAs in different species remains a mystery. Recent studies have shown that many lncRNAs can regulate gene expression during and after transcription, which has a significant impact on the development and progression of neurological disorders [170,180,181].

In addition, lncRNAs that can bind to transcription factors (TFs) actively participate in the regulation of gene expression by attracting TFs and directing them to specific regions in the DNA sequence, such as the promoter region, thereby controlling transcriptional activity. Additionally, another way of regulation involves the binding of multiple transcription factors to lncRNA molecules. In situations where multiple signaling pathways are activated simultaneously, downstream effector molecules can bind to the same lncRNA, facilitating convergence and integration of information between different signaling pathways.

Several studies have shown that many lncRNAs play an important role as mediators in brain development [182]. This can be seen in the inhibitory effect of lncRNA-GAS5 on the polarization of M2 microglia in the brain, leading to faster demyelination [181]. Maintaining neuronal cell identity, their plasticity, and stress response seem to be key functions of lncRNAs in brain development [183,184]. The expression of lncRNAs in the brain is localized in specific regions [185].

Many lncRNAs relevant to cognitive function have been identified in animal or cell studies [186].

Studies have confirmed the presence of regional and cell-type-specific expression patterns of lncRNAs in cognitively relevant memory brain regions such as the hippocampus, prefrontal cortex, and amygdala [186]. For example, lncRNA Gm9968 is particularly abundant in the mouse hippocampus and plays a significant role in diseases such as AD or epilepsy. lncRNA Rian can down-regulate *LIMK1* expression via negative regulation of miR143-3p. So, it is hypothesized that controlling the modulation of the lncRNA Rian/miR143-3p/*LIMK1* axis can improve cognitive problems after sevoflurane anesthesia [187].

Various ncRNA species function within the network and can affect gene expression. Published data indicate that miRNAs can alter gene activity by blocking translation or causing mRNA degradation. On the other hand, lncRNAs can control mRNA expression and degradation by competing with restricted miRNAs, known as ceRNAs. ceRNAs can control gene expression levels by competing with mRNAs for the same binding sites for microRNAs (miRNA response elements; MREs). Both compete to bind to miRNAs and control each other, forming ceRNA networks (ceRNETs). It has been observed that ceRNA networks associated with lncRNAs play a key role in synaptic plasticity, memory, and the regulation of amyloid-β-induced neuroinflammatory diseases [188].

ncRNA networks became a hot topic in neuroscience because noncoding RNAs constitute the majority of the organism’s transcriptome, which is abundant in the central nervous system and is intensively involved in the regulation of RNA transcription, and thus is associated with the mechanisms of many neurological symptoms and diseases [189]. For example, a circRNA-related ceRNA network has been shown to be mainly involved in dendritic development and memory (Sorbs2) and mouse neurodevelopment (ALS2), providing new ideas for the clinical diagnosis and treatment of AD [190]. The currently available methodology allows for the study of ncRNA–miRNA–mRNA networks of whole transcriptome profiles in selected tissue.

Wang et al. [191] published the first results of mRNA\circRNA\lncRNA expression analyses, protein–protein interaction (PPI) expression networks, and ncRNA-related ceRNA networks and ncRNA/mRNA coexpression networks in the hippocampus of the txJ mouse model of WD and indicated differentially expressed circRNAs, lncRNAs, and mRNAs that are associated with the mechanisms by which cognitive impairment occurs in WD.

In detail, the expression profiles of lncRNA, circRNA, and mRNA in the hippocampal tissue of txJ mice were investigated to identify differentially expressed RNAs (DE-RNAs). These DE-RNAs were then used to construct PPI networks as well as ceRNA networks encompassing DE-circRNAs and lncRNAs, alongside coding-noncoding coexpression (CNC) networks. To gain insight into their roles and pathways, gene ontology (GO) and Kyoto Encyclopedia of Genes and Genomes (KEGG) analyses were applied to the PPI and ceRNA networks.

Authors identified 361 DE-mRNAs (193 up-regulated and 168 down-regulated), 2627 DE-lncRNAs (1270 up-regulated and 1357 down-regulated), and 99 DE-circRNAs (68 up-regulated and 31 down-regulated) in txJ mice relative to controls.

Several mRNAs targeted by miRNAs in ncRNA-related ceRNA networks were also differentially expressed in this study, including *Fosb*, *Shank3*, *Cacna1i*, and others.

*Fosb* is an activity-dependent TF that is essential for hippocampal memory regulation and the *Fosb* family affects numerous addiction-related genes through histone modification. *Fosb* overexpression in the hippocampus can influence learning and memory processes. In mouse models of AD with cognitive dysfunction, *Fosb* has been shown to potentially inhibit *c-Fos* expression (an early gene vital for plasticity and cognition) by binding to promoters and triggering histone deacetylation. Due to *Fosb*’s long half-life, it may contribute to prolonged cognitive impairments.

As an excitatory postsynaptic scaffolding protein, SHANK3 interacts with various postsynaptic density proteins, regulating neurotransmitter receptors and signaling molecules. Research on autism spectrum disorder (ASD) suggests that variations in *SHANK3* mRNA levels can impact hippocampal synaptic transmission in mice, potentially leading to long-term deficits in learning and memory.

The *CACNA1I* gene, encoding the voltage-gated calcium channel Cav3.3, is considered a risk factor for schizophrenia. Variants in *CACNA1I* can disrupt neuronal excitability and brain network function, thereby affecting transmitter release, sensory processing, memory, and sleep.

A profile of circRNAs was also identified in the hippocampal tissue of TX mice with 99 significant differentially expressed circRNAs (DECs) selected. Notably, *mmu_circ_0001859* and *mmu_circ_0000242* were highly associated with multiple biological pathways, including axon guidance, Wnt Signaling, calcium signaling, mitogen-activated protein kinase (MAPK) signaling, focal adhesion, neurotrophin signaling, transforming growth factor beta (TGF-β) signaling, the cell cycle, ErbB signaling, notch signaling, p53 signaling, and Fc gamma R-mediated phagocytosis, among others.

The GO and KEGG analyses on both up- and down-regulated genes of circRNA-associated ceRNA networks respectively allowed us to identify a series of enriched terms associated with neurological diseases and cognitive processes.

The GO analysis for up-regulated genes showed enrichment in categories such as the cytoplasm (GO:0005737), protein binding (GO:0005515), nucleus (GO:0005634), and metal ion binding (GO:0046872). Meanwhile, KEGG pathway analysis indicated enrichment in pathways like Ras signaling (mmu04014), PI3K-Akt signaling (mmu04151), and calcium signaling (mmu04020). In contrast, GO analysis for down-regulated genes was enriched in areas like cell junctions (GO:0030054), cell projections (GO:0042995), synapses (GO:0045202), and cytoplasmic vesicles (GO:0031410). KEGG pathways for down-regulated genes included amphetamine addiction (mmu05031), adrenergic signaling in cardiomyocytes (mmu04261), tight junctions (mmu04530), and circadian entrainment (mmu04713).

Several ceRNA networks were identified as potentially involved in the cognitive impairment progression observed in WD. For instance, in the network centered on mmu-miR-6931-5p with *Fosb* as a target gene, 44 upstream circRNAs were identified that might act as sponges for mmu-miR-6931-5p, influencing the regulation of *Fosb* expression.

In the hippocampal tissue of txJ mice, 2627 differentially expressed lncRNAs (DELs) were identified, with functional enrichment analyses conducted on associated lncRNA-mediated ceRNA networks. Among these, GO terms for up-regulated target genes were enriched in processes such as cytokine production regulation (GO:0001817), xenobiotic metabolism (GO:0006805), ubiquitin ligase complex (GO:0000151), late endosome activity (GO:0005770), ubiquitin-conjugating enzyme activity (GO:0061631), and transcription corepressor activity (GO:0003714). KEGG pathways highlighted ubiquitin-mediated proteolysis (mmu04120) and multiple neurodegenerative pathways (mmu05022).

For down-regulated target genes, GO analyses indicated enrichment in oxidoreductase activity (GO:0016491), calcium ion binding (GO:0005509), microtubule-related functions (GO:0007018), and cellular structures like the apical plasma membrane (GO:0016324) and cilia (GO:0005929). KEGG analysis linked these down-regulated genes to neurodegenerative pathways (mmu05022), including Huntington’s disease (mmu05016), amyotrophic lateral sclerosis (mmu05014), and neuroactive ligand-receptor interactions (mmu04080).

One example within this ceRNA network is the targeting of *SHANK3*, where the lncRNA NONMMUT015424.2 potentially regulates *SHANK3* expression through sponging mmu-miR-5126. This predicted ncRNA–ceRNA interaction network may play a role in the cognitive impairments observed in WD, warranting further experimental validation to confirm these regulatory mechanisms.

The second study, which also analyzed changes in ncRNA expression in a mouse model of WD, used high-throughput transcriptome sequencing to assess lncRNA expression in the lentiform nucleus region [192]. The aim of this work was to identify novel lncRNAs and determine their involvement in WD-related networks.

In this study, 212 lncRNAs were differentially expressed, with 98 showing up-regulation and 114 down-regulation. Additionally, 32 mRNAs displayed differential expression, with 15 up-regulated and 17 down-regulated. Through Pearson correlation analysis and lncRNA-targeted miRNA-mRNA network predictions, 1131 coexpressed lncRNA-mRNA pairs were identified.

The differentially expressed lncRNAs were linked to various biological functions and signaling pathways, including translational initiation, motor learning, locomotor behavior, dioxygenase activity, postsynaptic membrane components, neuroactive ligand–receptor interaction, NF-κB signaling, cholinergic synapse, sphingolipid signaling, and Parkinson’s disease pathways, as shown by the GO and KEGG analyses.

RT-qPCR analysis confirmed significant expression differences in six lncRNAs (XR_001782921.1, XR_001780581.1, ENSMUST_00000207119, XR_865512.2, TCONS_00005916, and TCONS_00020683) between the TxJ mice and control group. Of these, four lncRNAs (TCONS_00020683, XR_865512.2, XR_001780581.1, and ENSMUST00000207119) exhibited high conservation, suggesting their potential relevance to WD pathogenesis.

This study identified Sp6, Mas1, Egr3, Adora2a, and Hoxb3 mRNAs as significant biomarkers for WD, providing insights into how these genes may contribute to WD symptoms. The *sp6* gene is crucial for cell proliferation during early development, particularly within germ layers, and later plays a role in the development of enamel, epidermis, and neural structures [193,194]. Recent research links *Sp6* expression in the amygdala and hippocampus to cognitive functions and motor disabilities, both of which are relevant to WD pathology [195]. Additionally, it supports the formation of the eye’s pigment cup, potentially linking it to WD corneal abnormalities [196]. *Mas1* is expressed in microglia and is primarily associated with anti-inflammatory signaling and with the renin–angiotensin system [197,198]. It can influence the NF-κB pathway, essential for neuronal survival, apoptosis, and synaptic plasticity [199,200]. Copper accumulation in the brain, characteristic of WD, can cause neuroinflammation and microglial damage. Therefore, *Mas1* may play a role in WD-related inflammation and neurodegenerative processes. *Egr3* is critical for the development of the sympathetic nervous system and plays a role in motor coordination and skill learning, functions often impaired in WD [201,202]. Adora2a is known to regulate neurotransmitter release. *Adora2a* gene polymorphisms have been associated with disorders like attention-deficit hyperactivity disorder (ADHD) and Tourette’s [203,204]. In the central nervous system, its activation can lead to neuronal damage and affect white matter—a common site of brain injury in WD [205]. Hoxb3 is a member of the Hox gene family. *Hoxb3* is highly expressed in early neural stem cells, influencing neuron proliferation and differentiation. It is also implicated in oligodendrocyte progenitor cell apoptosis in microglia [206], relevant to WD’s neurodegenerative effects.

GO analysis identified enriched biological functions like motor learning, locomotor behavior, and dioxygenase activity, aligning with WD’s neurological symptoms such as dyskinesia and bradykinesia, facial grimacing, dystonia, tremor, urinary incontinence, hyperreflexia, cognitive impairment, and other symptoms.

Thus, the biological functions identified through GO analysis exhibit a strong correlation with the clinical picture of WD, thus warranting further investigation into the lncRNAs and mRNAs associated with these biological functions.

The KEGG analysis identified notable enrichment in pathways including NF-κB, cholinergic synapse, sphingolipid metabolism, and Parkinson’s disease signaling. NF-κB is a key transcription factor involved in inflammation and immune responses. Beyond immunity, NF-κB also regulates development, cell growth, survival, and proliferation, linking it to various disease processes [207].

Studies show a strong link between the inflammatory response and the progression of WD, with high levels of inflammatory cytokines observed in WD patients, affecting their clinical symptoms. Studies on the txJ mice model showed the accumulation of copper in the striatum, accompanied by an increase in inflammatory response within the striatum and corpus callosum [146]. Consequently, alterations in synaptosomes, particularly in numerous synaptic proteins, occurred, thereby influencing motor symptoms.

Despite the numerous weaknesses of these studies, related to the small sample size, the possibility of differences in the transcriptional profile between animals and patients with different forms of WD or the analysis of only selected tissues, as well as the lack of experimental validation, including genes with significant expression differences and predictive network models, their results shed light on the expression profiles of lncRNAs, circRNAs, and mRNAs in hippocampal/brain lenticular nucleus tissues of txJ mice, providing valuable information on the functions of regulatory genes in WD associated with cognitive deficits. These findings may contribute to the development of diagnostic and therapeutic approaches for WD in the future. However, the mechanism of regulation of the expression of transcription molecules is complex and requires further studies, which should be directed, among other things, at identifying the regulation of epigenetic modifications.

## 4. Study Limitations

This narrative review discussing psychiatric symptoms of WD, their etiologies, and the broader influence of copper metabolism disorders on psychiatric manifestations in general psychiatry has certain limitations. Firstly, a consistent diagnostic approach across the studies reviewed was lacking, as the international diagnostic criteria for WD, such as the Leipzig score established in 2003 [208], have not been universally adopted. Furthermore, advancements in molecular diagnostics, particularly with next-generation sequencing (NGS), have become standard practice only recently, meaning that earlier studies primarily relied on clinical observations and biochemical indicators of copper metabolism disturbances [121].

Regarding copper metabolism biomarkers, there remains inconsistency in methodologies for assessing copper status. While total serum copper has been commonly used, recent studies have aimed to measure non-ceruloplasmin-bound copper, referred to as “directly analyzed non-ceruloplasmin-bound copper” (dNCC), along with labile or exchangeable copper [209]. These measurements may better represent the biologically active, toxic copper fraction. Establishing a standardized method for quantifying this free copper in circulation is therefore essential.

Additionally, this review focused exclusively on English-language publications, potentially omitting relevant findings published in other languages. Although these limitations could introduce bias, this work seeks to provide an overview based on the currently available data within international databases.

## 5. Conclusions

The mechanisms underlying psychiatric symptoms in Wilson’s disease (WD) are not yet fully understood. It has been suggested that these symptoms may result from the impact of the disease on daily life, including functional impairment, social and economic challenges, and related stressors. Many patients with WD experience adjustment disorders such as anxiety, depression, and behavioral problems. Although depression is common in other chronic diseases with similar levels of disability, its prevalence in WD is notably higher and more consistent with other BG disorders, suggesting that BG damage may be a contributing factor.

Thus, it is highly probable that the cause of the occurrence of depression as well as other psychiatric syndromes in WD may be damage to the BG. As in the CNS, WD mainly affects the BG—it may be hypothesized that the pathogenesis of neuropsychiatric symptoms may be similar to those seen in other neurodegenerative disorders that involve the BG (e.g., Parkinson’s disease [PD] or Huntington’s disease [HD]. Common neurotransmitter systems (dopaminergic, noradrenergic, and serotonergic) may underlie similar psychiatric symptoms in BG disorders, which may provide a basis for pharmacological treatment.

Cognitive deficits in WD (e.g., reduced processing speed, impaired working memory, and attention) may be related to changes in the cortico–striatal pathways and correlate with the global intensity of MRI abnormalities.

In addition, in WD—in the case of liver involvement—hepatic encephalopathy may exacerbate psychiatric symptoms, which should be considered when planning treatment, especially for drugs metabolized in the liver.

Copper, critical for proper CNS functions, has been implicated in various psychiatric symptoms when dysregulated. Possible mechanisms include direct copper toxicity, neurotransmitter imbalances, oxidative stress, mitochondrial dysfunction, cuproptosis, ferroptosis, mitophagy, neurotrophic factor deficiencies, *ATP7B* mutations, which potentially contribute to psychiatric symptoms in WD.

However, current evidence is insufficient to definitively establish the cause of psychiatric symptoms in WD. It is possible that the etiology of psychiatric symptoms varies among individuals, with multiple biological and psychological mechanisms contributing to them simultaneously. Future studies with larger samples and comprehensive analyses of different possible mechanisms may provide clearer insight into the causes of psychiatric symptoms in WD. Advances in genomic and transcriptome technologies as well as molecular modeling can provide valuable clinical information indicating new molecular pathways involved in the pathogenesis of mental disorders in WD, which may contribute to the improvement or development of new effective diagnostic or therapeutic methods. Further studies of genotype–phenotype relationships in WD, concerning mutations in the *ATP7B* gene as well as other potential modifying genes may also be important.

## Figures and Tables

**Table 1:** The frequency of various psychiatric symptoms in WD.

Scheme	Study Population	Country of Origin	Study Design
**Depression**			
Akil, 1991 [12]—27% depression	41	USA	retrospective
Dening, 1990 [36]—15% depression	129	UK	prospective
Rathbun, 1996 [29]—26% depression	34	USA	cross sectional
Shanmugiah, 2008 [30]—4% major depressive disorder, 2% dysthymia	50	India	cross sectional
Svetel, 2009 [31]—36% depression	50	Serbia	cross sectional
Mercier-Jacquerier, 2011 [32]—15.7% depression	19	France	retrospective
Carta, 2012 [33]—47.8% major depressive disorder	23	Italy	case control
Camarata, 2023 [19]—37% major depressive disorder	62	UK, USA	retrospective, cross sectional
**Mania, Bipolar Disorder**			
Oder, 1991 [14]—26.6% mood symptoms, 31.1% affective instability	45	Austria	prospective
Srinivas, 2008 [34]—2.5% bipolar disorder	15	India	retrospective
Shanmugiah, 2008 [30]—18% bipolar disorder	50	India	cross sectional
Carta, 2012 [33]—30% bipolar disorder, 39% mania/hypomania	23	Italy	case control
Camarata, 2023 [19]—9.8% bipolar disorder	62	UK, USA	retrospective, cross sectional
**Anxiety**			
Akil, 1991 [12]—8% anxiety	41	USA	retrospective
Rathbun, 1996 [29]—20% anxiety	34	USA	cross sectional
Svetel, 2009 [31]—62% anxiety	50	Serbia	cross sectional
Carta, 2012 [32]—8.7% panic disorder	23	Italy	case control
Camarata, 2023 [19]—30.6% anxiety, 8.1% panic disorder, 8.1% generalized anxiety disorder, 8.1% agoraphobia	62	UK, USA	retrospective, cross sectional
**Psychosis**			
Oder, 1991 [14]—6.7% psychosis	45	Austria	prospective
Huang, 1992 [34]—11.3% schizophrenia	71	Taiwan	retrospective
Rathbun, 1996 [29]—2% psychosis	34	USA	cross sectional
Srinivas, 2008 [34]—1.4% schizophrenia, 0.8% schizoaffective disorder	15	India	retrospective
Camarata, 2023 [19]—1.6% psychosis	62	UK, USA	retrospective, cross sectional
**Personality Changes**			
Dening, 1990 [36]—15% irritability	129	UK	prospective
Akil, 1991 [12]—45.9% personality changes	41	USA	retrospective
Huang, 1992 [35]—38% personality changes	71	Taiwan	retrospective
Svetel, 2009 [31]—26% irritability, 24% disinhibition, 24% apathy	50	Serbia	cross sectional
Mercier-Jacquerier, 2011 [32]—two cases of impulsivity	19	France	retrospective
Cognitive impairment			
Akil, 1991 [12]—10.8% cognitive symptoms	41	USA	retrospective
Camarata, 2023 [19]—36.7% cognitive symptoms	62	UK, USA	retrospective, cross sectional
**Sleep disturbances**			
Nevsimalova, 2011 [37]—27.5% poor nocturnal sleep (lower sleep duration, decreased sleep efficiency, increased wakefulness), 70% daytime sleepiness, 27% restless legs syndrome	55	Czech Republic	cross sectional
Tribl, 2016 [38]—12.2% REM sleep behavior disorder	41	Brasil	prospective
**Other**			
Akil, 1991 [12]—5% catatonia	41	USA	retrospective
Oder, 1991 [25]—15.5% past suicide attempts, 20% impaired social judgment, 24.4% belligerence	45	Austria	prospective
Mercier-Jacquerier, 2011 [32]—one case of anorexia nervosa	19	France	retrospective
Nevsimalova, 2011 [37]—24.1% cataplexy	55	Czech Republic	cross sectional
Camarata, 2023 [19]—4.8% posttraumatic stress disorder, 8.1% obsessive compulsive disorder, 3.2% substance use disorder, 6.5% alcohol use disorder	62	UK, USA	retrospective, cross sectional
